# Shape of the mitral annulus in normal individuals and dilated cardiomyopathies: computational modeling insights into leaflet stress distribution

**DOI:** 10.3389/fphys.2025.1532972

**Published:** 2025-03-25

**Authors:** Salvatore Pasta, Eluisa La Franca, Fabrizio Crascì, Giovanni Gentile, Manlio Cipriani, Francesco Fulvio Faletra

**Affiliations:** ^1^ Department of Engineering, Università degli Studi di Palermo, Palermo, Italy; ^2^ Department of Research, IRCCS ISMETT, Palermo, Italy; ^3^ Department for the Treatment and Study of Cardiothoracic Diseases and Cardiothoracic Transplantation, IRCCS-ISMETT (Istituto Mediterraneo per i Trapianti e Terapie ad alta Specializzazione), Palermo, Italy; ^4^ Radiology Unit, IRCCS-ISMETT, Palermo, Italy

**Keywords:** mitral valve, saddle-shaped mitral annulus, planar mitral annulus, computational modeling, mitral annulus disjunction

## Abstract

**Introduction:** The mitral valve annulus naturally adopts a saddle shape in systole, likely concentrating systolic stress on the commissures where fibrous trigones are located. This study hypothesized that in patients with dilated cardiomyopathies, where the annulus is large and planar, the stress would be redirected.

**Methods:** Computational modeling was employed to compare the stress distribution in saddle-shaped mitral valves (n.10 patients) with planar annuli seen in dilated cardiomyopathy (n.10 patients) using kinematics of the mitral valve annulus from systole to diastole extrapolated from computed tomography angiography.

**Results:** Simulations revealed high stress near the anterolateral and posteromedial commissures in normal valves, in contrast to high leaflet stress in planar annuli. Significant differences in stress distribution were observed near the anterolateral (S = 0.427 ± 0.053 MPa in normal valves vs S = 0.211 ± 0.123 MPa in diseased valves, p < 0.001) and posterolateral commissures (S = 0.340 ± 0.008 MPa in normal valves vs S = 0.208 ± 0.060 MPa in diseased valves, p < 0.001). Additionally, mitral annulus disjunction was present in healthy patients but absent in those with annulus planarity due to dilated cardiomyopathy.

**Discussion:** This study suggests that while the saddle-shaped annulus focuses leaflet stress on commissures, planar annuli distribute systolic stress over leaflet surfaces. This may trigger embryonic pathways and alter mitral leaflet collagen content, ultimately leading to valve remodeling. Identifying patients with early annular planarity prior to substantial leaflet remodeling may provide early treatments to prevent increasing mitral regurgitation.

## 1 Introduction

The three-dimensional (3D) saddle-shaped arrangement of the mitral annulus was first described by Robert Levine in 1987 (Levine et al., 1989). This study triggered great enthusiasm and had the merit of generating a profound conceptual reconsideration of the echocardiographic criteria of mitral valve prolapse. Since the load on mitral leaflets is determined by the pressure gradient between the left ventricle and atrium, leaflet area, and leaflet curvature (Kunzelman et al., 1993a), Salgo et al. (2002) speculated that the non-planarity of the annulus may minimize the stress on the leaflets by increasing the natural leaflet systolic curvature. The saddle shape of the annulus appears to be a common feature among large mammals, configuring the hypothesis that this anatomical arrangement could be an advantageous adaptive mechanism that evolved over millions of years (Salgo et al., 2002). Though not described in the manuscript, the stress distribution on the mitral valve leaflets illustrated in Figure 6 of Salgo’s work (Salgo et al., 2002) caught our attention: the saddle-shaped annulus appears to focus systolic stress on the commissures in the same regions where anatomists describe the fibrous trigones. It may be speculated that, over millions of years, the gradual changes in the annular shape from planar to non-planar were accompanied by the appearance and gradual growth in size and consistency of these fibrous nodules in the regions where they would counteract increased stress. Our reasoning is as follows: If it is true that the non-planar annulus focuses stress on the commissure, then in a large, planar annulus (i.e., as in dilated cardiomyopathies), the stress should be diverted elsewhere, for instance, to the leaflet surface.

Extensive research has been performed on mitral valve modeling to enhance the understanding of the severity of the valvular disease. Finite-element analysis revealed that when the mitral valve annulus is dilated and exhibits reduced dynamics, complete coaptation is unattainable (Kunzelman et al., 1997). In another study, the mitral valve was modeled in an unloaded configuration, incorporating time-dependent kinematic boundary conditions to simulate the movement of the mitral annulus and papillary muscles during valve closure (Stevanella et al., 2009b). Recently, research groups have concentrated on mitral valve repair techniques, including edge-to-edge repair with Mitraclip (Pasta, 2023; Sun et al., 2014), annuloplasty band rings (Baillargeon et al., 2015), and transcatheter devices (Catalano et al., 2023). The application of fluid-solid interaction techniques has also been proposed to account for flow-induced hemodynamics of the mitral valve (Christierson et al., 2024; Dahl et al., 2012).

To test this hypothesis, we employed a combination of computed tomography angiography (angio-CT) imaging and computational modeling to quantify the stress distribution in mitral valves with morphologically normal mitral annuli and those with diseased annuli. Angio-CT with voxel size 0.6 mm was preferred for its superior spatial resolution over echocardiography, facilitating the acquisition of a realistic anatomy of the mitral valve. Cardiac phases extrapolated from the angio-CT imaging of each patient were used to determine the annulus displacements required for computational predictions, thus enabling the modeling of actual mitral valve kinematics. The study group comprising healthy patients aimed to investigate the impact of the saddle-shaped arrangement of the mitral annulus on stress distribution, whereas patients with dilated cardiomyopathies were included to explore stress in the planar annulus structure. By directly comparing the resulting stress distribution of mitral valve leaflets between the saddle-shaped and planar mitral valves, we could delve into the mechanics of mitral annulus.

## 2 Methods

### 2.1 Mitral valve model

**FIGURE 1:**
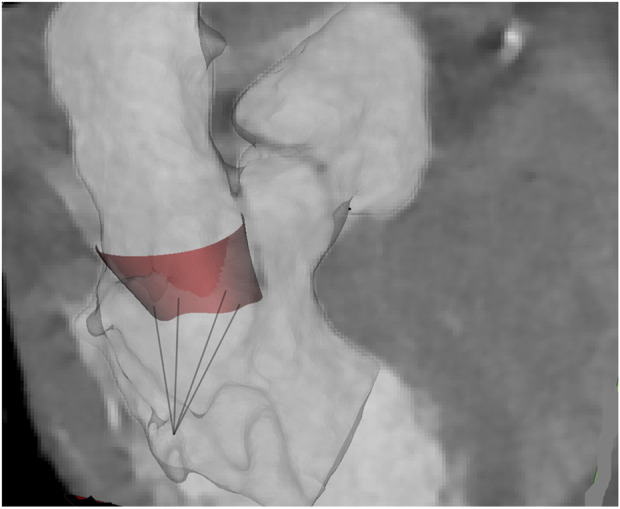
Representative shape of MV and chordae at atrial systole as superimposed onto the original angio-CT images.

Ten patients with morphologically normal mitral valves and ten patients with non-ischemic dilated cardiomyopathy were retrospectively collected from angio-CT imaging scans (see Table 1). Healthy patients did not have any sign of mitral regurgitation while only two patients with cardiomyopathy had regurgitation graded as moderate. Diagnostic images had an isotropic voxel size of 0.6 mm with slice thickness of 0.625 mm. Utilizing Mimics medical imaging software (v21, Materialise, BE), the end-systolic configuration was processed to generate the valve surface model. Specifically, the contour of the saddle-shaped annulus was manually segmented using a spline curve at end-systole. The mitral valve annulus at end-diastole was also segmented as this information was necessary to quantify annulus kinematics. To define the free edge of the anterior and posterior mitral valve leaflets, an additional spline curve was generated based on the contour of the black end of the leaflet free margin. Finally, the leaflet surface was developed by loft protrusion of the spline curves defining the annulus and leaflet free margin in the Rhinoceros CAD modeling software (v7, McNeel, United States of America). Papillary muscle positions were identified at both end-systole and end-diastole to track chordae tendineae motion throughout the cardiac cycle. These changes in the position of papillary muscles were utilized as input in the computational model. Two uniform sets of chordae tendineae were generated as lines between papillary muscles and the mitral valve model using several random points distributed along leaflet margins (Stevanella et al., 2009a). Figure 1 illustrates a representative image of the mitral valve geometry superimposed onto the original angio-CT image. For each patient, mitral annulus dysfunction was measured at the systolic phase after reformatting the ECG-gated CT scan. The study was approved by our institutional review board (IRRB/04/14), and all patients gave informed consent prior to study enrolment.

**TABLE 1:** Demographic data and measurement of MAD at imaging analysis; data are shown as mean and standard deviation.

	Healthy	Cardiomyopathy
Age (yrs)	56.8 ± 15.7	61.0 ± 16.4
Male (%)	75.5	87.0
BSA (m^2^)	1.9 ± 0.2	2.0 ± 0.2
LV Wall (mm)	4.9 ± 0.2	5.4 ± 0.4
EF (%)	59.1 ± 4.4	23.4 ± 14.6 *
V_ES_ (mL)	124.1 ± 18.4	186.3 ± 34.3
V_ED_ (mL)	50.4 ± 8.1	238.0 ± 40.2 *
MAD (mm)	3.8 ± 1.1	—
MA Diameter Long (mm)	42.4 ± 5.4	44.2 ± 6.4
MA Diameter Short (mm)	23.3 ± 6.5	40.2 ± 5.9 *

Note: BSA, body surface area; LV, Wall = left ventricular myocardial thickness; EF, ejection fraction; V_ES_, end-systolic left ventricular volume; V_ED_, end-diastolic left ventricular volume; * indicates statistically different from healthy subject.

### 2.2 Computational modeling

The mitral valve model underwent meshing using triangular shell elements with a size of 0.12 mm in the ABAQUS finite-element software (v2021hf7, Dassault Systemes, United States of America). Chordae were meshed using beam elements of size 0.1 mm, assuming a constant circular cross-sectional area of 0.412 mm^2^ for each chorda. To simulate the mitral valve behavior, the stress-strain response documented by May-Newman and Yin (1995) under biaxial loading was employed. This involved modeling the mitral valve with a hyperelastic, isotropic, incompressible material using Yeoh’s two-term relationship (Equation 1):

$$W=C_{1}\left(I_{1}-3\right)+C_{2}{\left(I_{1}-3\right)}^{2}$$ (1)

Where $C_{1}$ and $C_{2}$ C2 are material constants resulting from the biaxial fitting (i.e., C10 of 0.318 MPa and C20 of 0.836 MPa) and $I_{1}$ is the first deformation invariant.

Conversely, the constitutive relationship for the chordae utilized Marlow’s hyperelastic strain energy potential ( $W=W_{dev}\left(I1\right)$ derived from uniaxial test data from Kunzelman et al. (1993b). For all mitral valve models, the anterior leaflet was set at a thickness of 1.3 mm, while the posterior leaflet was assigned a thickness of 1.2 mm according to literature data (Stevanella et al., 2009a; Kunzelman et al., 1993b).

To account for the mitral annulus kinematics seen at angio-CT imaging, annular contraction was considered by applying nodal displacements to the element nodes within the annulus. These displacements were computed from segmented splines at both end-diastole and end-systole, tailored individually for each patient, and were imposed for the entire cardiac cycle. Displacement boundary conditions were also applied to the papillary muscles to account for left ventricular beating. Simulation of valve closure involved applying a time-dependent physiologic transmural pressure to the mitral valve leaflet surface facing the left ventricle (Lau et al., 2010). The duration of the transmural pressure curve was scaled for each patient using the collected heartbeat during clinical assessments. A general contact algorithm simulated leaflet contact, while tie contact conditions were established between the chordae and the free edges of the leaflets.

Assessment of mitral valve biomechanics involved evaluating maximum principal stress computed across various anatomic quadrants. Statistical analysis utilized one-way ANOVA, followed by Holm-Sidak *post hoc* tests, to compare stress distribution among quadrants, setting significance at α = 0.05.

## 3 Results

**FIGURE 2:**
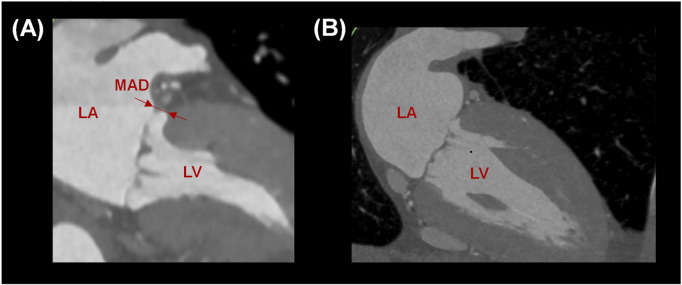
**(A)** Measurement of MAD in a healthy subject and **(B)** absence of MAD in a patient with planar annulus.

After reformatting the angio-CT scans, the presence of mitral annulus disjunction (MAD) was quantified by an expert radiologist (see Figure 2). In healthy subjects, the disjunction of mitral valve leaflets was predominately present at both the anterolateral side (P1 for n = 4) and posterolateral side (P3 for n = 6) of the posterior leaflets in continuity with fibrous trigones. The mean value of MAD was 3.8 ± 1.1 mm, shown in Table 1. On the contrary, no disjunction was observed in patients with dilated cardiomyopathy except for one patient.

**FIGURE 3:**
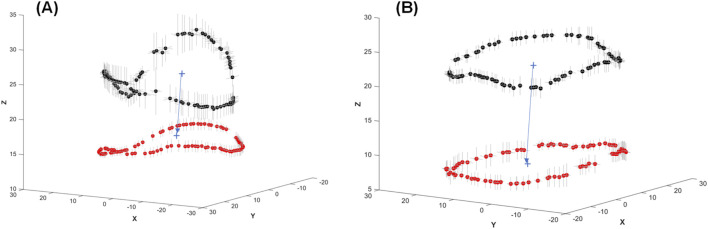
Mean values of the mitral annulus shape at ventricular diastole (black dots) and ventricular systole (red dots) showing the motion of the annulus for **(A)** healthy and **(B)** diseased mitral valves. Grey lines in the X and Y directions represent the mean standard error of mitral annulus displacement among the patients.

From the medical imaging analysis, the mean shape of the mitral annulus during ventricular systole and diastole was calculated for both healthy patients and those with annulus planarity. Subsequently, the position of the mitral annulus and its variation (i.e., mean standard error) were plotted to visually quantify the dynamics of the mitral annulus (see Figure 3). This was accomplished by first extracting fifty data points from each annulus and then aligning these points using the iterative closest point registration algorithm. In healthy patients, the mitral annulus demonstrated a saddle-shaped configuration during atrial diastole and was characterized by a downward expansion in systole. Conversely, patients with annulus planarity due to cardiomyopathy displayed a flattened conformation of the mitral annulus, paradoxically expanding during atrial systole with less distension compared to healthy patients.

**FIGURE 4:**
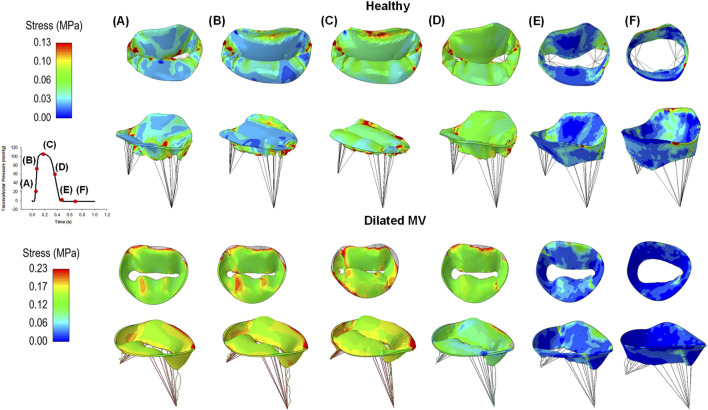
Colored maps of maximum principal stress of healthy (first row) and diseased mitral valves (second row) at different steps of the cardiac cycle **(A-F)**; the red color indicates the region of mitral valve leaflets at high stress while the blue color represents regions of low stress magnitude.

As the transmural pressure increased during valve closure, high stress was observed in the mitral valve leaflets, peaking when the valve reached ventricular systole (Figure 4). Stress release was evident as the transmural pressure decreased during ventricular diastole. In healthy individuals, the saddle-shaped mitral annulus led to stress accumulation near the trigones, a phenomenon not observed in patients with planarity of the mitral valve annulus. Indeed, local stress maxima occurred near the anterolateral and posteromedial commissures in the morphologically normal mitral valves. Conversely, patients with a planar annulus exhibited high stress throughout the whole valve.

**FIGURE 5:**
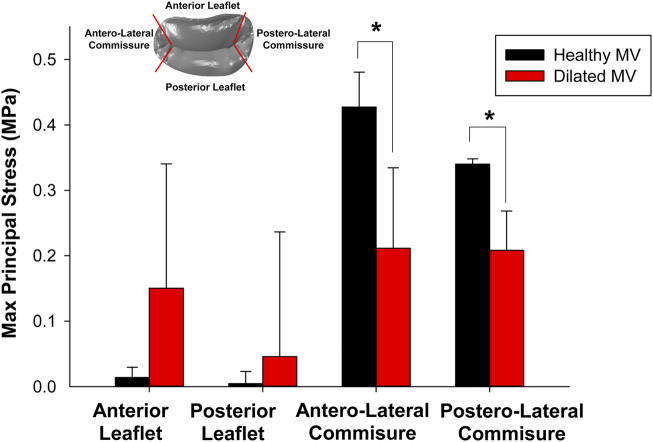
Comparison of maximum principal stress computed at peak systole among different quadrants for healthy and diseased mitral valves.

One-way ANOVA analysis revealed significant differences in the maximum principal stress (S) among segments of healthy mitral valves, as illustrated in Figure 5. Stress values were extracted through the ABAQUS graphical user interface using the probe command, followed by statistical analysis. Specifically, stress values near the anterolateral and posteromedial commissures of the saddle-shaped mitral annulus were statistically higher than those observed in corresponding anterior and posterior leaflets (refer to black bars alone). In contrast, there was no statistically significant difference in the stress magnitudes among quadrants for dilated mitral valves with a planar annulus (refer to red bars alone). Most importantly, a statistical difference was observed in the stress computed for the saddle-shaped annulus compared to that of the planar annulus (i.e., black *versus* red bars) near the anterolateral commissure (i.e., S = 0.427 ± 0.053 MPa for normal mitral valves and S = 0.211 ± 0.123 MPa for diseased mitral valves p < 0.001) and the posterolateral commissure (i.e., S = 0.340 ± 0.008 MPa for normal mitral valves and S = 0.208 ± 0.060 MPa for diseased mitral valves, p < 0.001).

**FIGURE 6:**
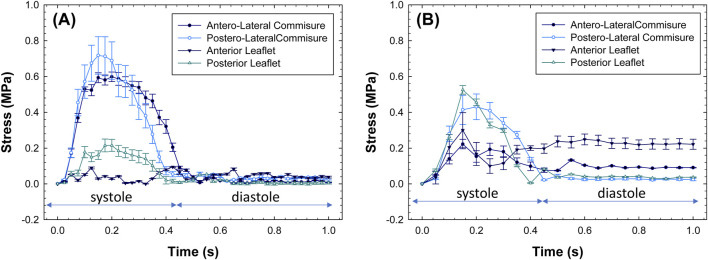
Stress values as a function of beat time simulation for **(A)** healthy and **(B)** diseased mitral valves.

Figure 6 shows the changes in stress magnitudes at different quadrants over the cardiac cycle. Post-processing was conducted using the ABAQUS graphical user interface to extract stress magnitudes at various time frames of the simulated cardiac cycle. For morphologically normal mitral valves, the region near the anterolateral and posterolateral commissures shows an increase in stress values upon closure of the mitral valve, followed by a stress release during diastole. However, the stress appears uniform among quadrants of diseased mitral valves during the whole cardiac beat.

## 4 Discussion

This study employed computational modeling to evaluate the biomechanical response of the mitral annulus dynamics in healthy subjects with a saddle-shaped annulus *versus* patients with dilated cardiomyopathy characterized by annulus planarity. The most notable observation involves the altered stress distribution patterns on the mitral valve leaflets in diseased patients compared to healthy subjects exhibiting a saddle-shaped mitral annulus. While in healthy subjects, the stress peaks were concentrated near the trigones when the valve closed, likely due to natural changes in the curvature of the saddle-shaped annulus during the cardiac cycle, in patients with dilated cardiomyopathy the stress was spread over the whole leaflet surface. From these results, two speculations were constructed:

First speculation: The long-held assertion that the MV leaflets are innocent bystanders in the pathophysiology of functional mitral regurgitation (FMR) in dilated cardiomyopathies was challenged several years ago by Dal-Bianco et al. (2009), who demonstrated that in FMR, leaflets are larger and thicker with cellular changes suggestive of reactivated embryonic pathways. They termed this phenomenon active “adaptation” of the valve, disrupting the paradigm that in FMR, leaflets and chordae are “structurally normal.” They also speculated that mechanical stress imposed on mitral leaflets tethered by papillary muscles was the trigger to reactivate embryonic pathways. We extended this observation by showing that, contrary to a non-planar annulus in which leaflets are spared from systolic stress, in a planar annulus, systolic stress is distributed over the entire surface of the MV leaflets. This may be an additional trigger for reactivating embryonic pathways and altering the mitral leaflet collagen content, eventually leading to an increased mitral leaflet length, area, and thickness.

Second speculation: Our study allows for further speculation using a new hypothesis on the bimodal distribution of MAD in normal individuals. It should be noted that, while MAD represents a separation between the ventricular myocardium and the mitral annulus, mitral valve prolapse is characterized by the displacement of one or both mitral valve leaflets. Although MAD can be observed in conjunction with valve prolapse, it can also occur independently. Toh et al. (2021) using CT scans observed that MAD is not limited to mitral valve prolapse as previously described in both anatomic and imaging studies (Drescher et al., 2022; Eriksson et al., 2005), but is also present in more than 90% of normally structured mitral valves. Drescher et al. (2022) indicated that the prevalence of MAD ranges from 16% to 55% among individuals with mitral valve prolapse and/or severe mitral regurgitation. Annulus disjunction can reach 96% in healthy people (Toh et al., 2021), but it is uncommon in patients with dilated cardiomyopathy. MAD represents an additional risk factor for invasive treatments and is associated to wall thinning. This mitral annulus disjunction is localized below the medial (P3) and lateral (P1) areas of the posterior mitral valve leaflet. In the present study, we corroborated findings from Toh et al. (2021) as healthy subjects presented disjunction of the mitral valve with different extensions under the medial and lateral scallops. This anatomic arrangement of mitral annulus disjunction has been described in autoptic samples by Angelini et al. (1988), who showed that the hinge line of the posterior leaflet is separated from the ventricular wall by a sub-annular membrane-like, or thicker, flap of fibrous tissue. McAlpine first described this membrane in one of his elegant dissections of a normal heart in 1976 (McAlpine, 1975). He used the term ‘‘subvalvular membrane’’ to define this collar-shaped fibrous extension found between the hinge of the posterior leaflet and the crest of the ventricular wall. However, neither Toh, Angelini, nor McAlpine gave a physiologic explanation for this peculiar anatomical arrangement. Importantly, McAlpine, and formerly Henle (Henle, 1876), presented mitral annulus disjunction in the context of morphologically normal heart structures, and this has been confirmed in subsequent studies (Toh et al., 2021; Chess et al., 2023). We maintain that this specific anatomic extension of MAD from the trigones towards the medial and lateral parts of the annulus could be simply considered a three-dimensional extension of the trigones to counteract the intraventricular loads exerted on the mitral leaflets. Though this hypothesis needs further “*ad hoc*” investigations, the disjunction of the mitral annulus in the atrial wall could be the result of a natural tissue adaptation governed by growth and remodeling principles. The principle of protecting the lateral commissures of mitral leaflets with a band of fibrous tissue may be considered equivalent to the principle of reinforcing the arterial wall with external support (Thubrikar et al., 1988). When the artery is encased in a rigid biomaterial, the wall stress resulting from blood pressure is transmitted from the compliant tissue wall to the rigid material, thus leaving the artery with low circumferential stress despite the high systemic pressure. This principle of reinforcement suggests that the surrounding rigid tissue withstands high stress while preserving the function of the more compliant mitral valve leaflets. Therefore, the fibrous rigid-like structure of trigones and the fibrous band surrounding the mitral annulus may act as a stiff tissue that withstands the high wall stress induced by cardiac contraction, thereby reducing the stress exerted on the mitral valve and ultimately preserving its function. It should be noted that MAD was not observed in patients with dilated cardiomyopathy where the stress analysis revealed maxima near the mitral valve leaflets. This could be the result of ventricular dilatation stretching the mitral valve annulus, likely flattening the fibrous subvalvular membrane near the trigones. In this setting, the stress accumulates more noticeably on mitral valve leaflets.

There are several limitations to this study. From a clinical perspective, the findings should be confined to the present study group, and validation on a larger patient dataset is necessary to extend our results. Patients with cardiomyopathy characterized by a planar mitral annulus were selected as the control group. In addition to potential biases in patient cohort demographics, various factors such as the definition of disjunction and measurement evaluations may have influenced the findings of this study. From a numerical standpoint, the presence of myocardial tissues, thus the trigones, were not considered in this study. While it is crucial to consider the myocardial tissue surrounding the mitral valve, our inclusion of the motion of the mitral annulus has allowed for an effective investigation of mitral annulus biomechanics. Furthermore, the mitral valve was simulated using an isotropic hyperelastic constitutive material, while other computational studies have adopted fiber-reinforced anisotropic models to simulate mitral valve biomechanics. A uniform material thickness was applied to both the mitral valve leaflets and chordae due to the lack of clinical data available to customize the model for each patient’s specific tissue thickness. To verify the assumption regarding material thickness, the extent of variation in stress distribution when changing leaflet thickness can be investigated. We recognize that our study focused on the structural mechanics of the mitral valve, excluding the dynamic effects of blood flow. Integrating hemodynamic factors like fluid shear forces may yield a more comprehensive understanding of stress distribution. Fluid-structure interaction simulations might explain the combined influences of blood flow and structural mechanics on mitral valve performance. This approach would improve the physiological relevance of computational models and potentially guide more effective therapeutic strategies. Further studies will be also performed to examine time-dependent changes in the curvature of the saddle-shaped annulus and assess the changes in stress distribution associated with the remodeling of mitral valve structure.

## 5 Conclusion

This study concluded that.

• In normal individuals, the saddle-shaped configuration of the annulus protects the leaflets by diverging systolic stress at the commissures. We speculate that the trigones and their extensions serve to counteract this systolic stress.
• The planarity of the annulus in dilated cardiomyopathies distributes systolic stress across the entire surface of the anterior and posterior leaflets. We speculate that this may be an additional trigger to reactivate the embryonic pathway, leading to an increase in the surface area and thickness of mitral leaflets in dilated cardiomyopathies.

## Data Availability

The raw data supporting the conclusions of this article will be made available by the authors, without undue reservation.
